# A Piscine Birnavirus Induces Inhibition of Protein Synthesis in CHSE-214 Cells Primarily through the Induction of eIF2α Phosphorylation

**DOI:** 10.3390/v7041987

**Published:** 2015-04-15

**Authors:** Amr A.A. Gamil, Stephen Mutoloki, Øystein Evensen

**Affiliations:** Faculty of Veterinary Medicine and Biosciences, Norwegian University of Life Sciences, P.O. Box 8146 Dep., 0033 Oslo, Norway; E-Mails: amr.gamil@nmbu.no (A.A.A.G.); stephen.mutoloki@nmbu.no (S.M.)

**Keywords:** Birnavirus, infectious pancreatic necrosis virus, eIF2α, inhibition of protein synthesis, Mx, apoptosis

## Abstract

Inhibition of protein synthesis represents one of the antiviral mechanisms employed by cells and it is also used by viruses for their own propagation. To what extent members of the *Birnaviridae* family employ such strategies is not well understood. Here we use a type-strain of the Aquabirnavirus, infectious pancreatic necrosis virus (IPNV), to investigate this phenomenon *in vitro*. CHSE-214 cells were infected with IPNV and at 3, 12, 24, and 48 hours post infection (hpi) before the cells were harvested and labeled with S^35^ methionine to assess protein synthesis. eIF2α phosphorylation was examined by Western blot while RT-qPCR was used to assess virus replication and the expression levels of IFN-α, Mx1 and PKR. Cellular responses to IPNV infection were assessed by DNA laddering, Caspase-3 assays and flow cytometry. The results show that the onset and kinetics of eIF2α phosphorylation was similar to that of protein synthesis inhibition as shown by metabolic labeling. Increased virus replication and virus protein formation was observed by 12 hpi, peaking at 24 hpi. Apoptosis was induced in a small fraction (1−2%) of IPNV-infected CHSE cells from 24 hpi while necrotic/late apoptotic cells increased from 10% by 24 hpi to 59% at 48 hpi, as shown by flow cytometry. These results were in accordance with a small decline in cell viability by 24hpi, dropping below 50% by 48 hpi. IPNV induced IFN-α mRNA upregulation by 24 hpi while no change was observed in the expression of Mx1 and PKR mRNA. Collectively, these findings show that IPNV induces inhibition of protein synthesis in CHSE cells through phosphorylation of eIF2α with minimal involvement of apoptosis. The anticipation is that protein inhibition is used by the virus to evade the host innate antiviral responses.

## 1. Introduction

Cells employ inhibition of protein synthesis as a defensive mechanism in response to virus invasion, the aim being to limit the production of virus progeny and consequently arrest the spread within the organism. In higher vertebrates, there are mainly three well-known mechanisms by which inhibition of protein synthesis is induced during virus infections: (1) through the activation of the interferon inducible, dsRNA-activated protein kinase R (PKR); (2) through the activation of the PKR-like endoplasmic reticulum (ER) kinase (PERK); and 3) through factors associated with the induction of apoptosis. PKR is activated and auto-phosphorylated upon recognition and binding of dsRNA to its binding motif in the N terminus [1]. Phosphorylated PKR in turn phosphorylates the eukaryotic initiation factor 2-alpha (eIF2α). eIF2α phosphorylation blocks eIF2B-mediated GDP−GTP exchange preventing the formation of GTP–eIF2–tRNAi^Met^ ternary complex, which is crucial for translation initiation, and results in translation inhibition [2]. eIF2α phosphorylation can also be induced through PERK which is activated due to the accumulation of unfolded protein in the ER lumen, as a result of high virus replication, leading to the initiation of what is known as the unfolded protein response (UPR) [3]. Apoptosis on the other hand is induced by many innate and adaptive antiviral mechanisms [4], thereby leading to the activation of intracellular caspases during the early stages of apoptosis. Some of the caspases executing apoptosis can target components of the translation machinery for proteolysis [5].

Viruses, as obligatory pathogens, depend on the host machinery to produce their own proteins. They have evolved different strategies of taking advantage of the host machinery of protein synthesis that may or may not be associated with inhibition of protein synthesis. Picorna- and rotaviruses break translational control by targeting and disrupting different components of the translational apparatus [6,7,8]. As a result, the cells lose control of the translation machinery leading to the inhibition of host protein synthesis [9,10]. Other viruses such as adeno- and herpesviruses disrupt the regulatory pathway that inhibits protein synthesis in response to virus infection through PKR activation [11,12]. For birnavirus infections, there are no studies that clearly demonstrate how inhibition of protein synthesis occurs and to whose benefit.

IPNV is a type species of the genus Aquabirnavirus. It is an icosahedral, non-enveloped, double-stranded RNA virus consisting of two segments [13]. The first segment (A) is 3,092 bp long and contains two overlapping open reading frames (ORFs): a short one encoding a non-structural protein VP5 [14,15] and a long ORF encoding structural proteins VP2 and VP3 as well as a non-structural protein VP4 [16,17]. Segment B is 2,784 bp long and contains only one ORF encoding the viral polymerase VP1 [18]. Early studies using a piscine birnavirus infectious pancreatic necrosis virus (IPNV) showed that the virus was not associated with inhibition of protein synthesis although it suppressed DNA synthesis in permissive host cells [19,20]. In another study characterizing eIF2α and its response to endoplasmic reticulum stress, IPNV phosphorylated eIF2α [21], suggesting that inhibition of protein synthesis is in fact induced although this was not the focus of the study. In a recent study, Chen *et al*. showed that a low virulent recombinant strain of IPNV is able to induce inhibition of protein synthesis in RTG-2 cells [22]. In the present study, we demonstrate not only that this virus induces inhibition of protein synthesis in CHSE-214 cells but also that the virus probably uses this mechanism to its advantage.

## 2. Materials and Methods

### 2.1. Cell Lines

Chinook salmon embryonic (CHSE-214) [23] and Asian Grouper strain K (AGK) [24] cells were maintained in L-15 media with Glutamax® (Gibco, Carlsbad, CA, USA) supplemented with 5% FBS (Sigma Aldrich, St. Louis, MO, USA). For maintenance, CHSE-214 cells were grown at 20 °C while AGK cells were kept at 28 °C.

### 2.2. Virus Propagation

A recombinant IPN virus (rNVI-15R^b^) previously produced by reverse genetics [25] was used. In order to obtain adequate amounts of virus for use in the inhibition of protein synthesis experiment, the virus was first inoculated into 70%–80% confluent AGK cells followed by incubation at 15 °C until full CPE. The supernatant containing the virus was then harvested and clarified by centrifugation at 2500 rpm for 10 min. The concentration of the virus was estimated by titration in 96-well plates (Falcon, Bedford, MA, USA) containing 80%–90% confluent CHSE cells.

### 2.3. Virus Infection and Metabolic Labeling

Six-well plates containing approximately 90% confluent CHSE cells were used. Inoculation of cells in wells was done sequentially, one well for each time point to yield cells infected either for 3, 12, 24 or 48 h by the time of sampling. The cells were infected at a multiplicity of infection (MOI) of 20 with the purpose of obtaining one-cycle infection kinetics, and the experiment was repeated four times.

Level of protein synthesis was evaluated by monitoring S^35^ methionine incorporation into proteins. Cells were washed 3× with PBS followed by incubation with Methionine, Lysine and L-glutamine free Dulbecco’s modified Eagle’s medium (sigma Aldrich) containing 20µCi/mL S^35^ Methionine (Hartmann analytic, Braunschweig, Germany), 1% L-glutamine (Sigma Aldrich) and 2% FBS for 30 min. Thereafter, the cells were washed once with PBS, lysed using 250 µL Cell M lysing reagent (Sigma Aldrich) and then slowly agitated for 15 min. Cells were then scraped from the wells and transferred to 1.5 mL Eppendorf tubes together with the supernatants. Lysates were centrifuged at 13,000 rpm for 5 min to remove the cell debris and nucleus. Finally, the supernatants were transferred to new Eppendorf tubes and kept at −80 °C until required.

To evaluate the level of protein synthesis, cell lysates were subjected to gel electrophoresis. 10 µg of total cell proteins from each sample was applied to a Nupage mini gel (Invitrogen, Carlsbad, CA, USA) and subjected to electrophoresis at 200V. The separated proteins were transferred from the gel to a PVDF membrane using a semi-dry blotter (Biorad, Hercules, CA, USA). The membrane was incubated in a storage phosphor cassette overnight. Finally, radioactivity was detected using the Typhoon (GE Healthcare, Piscataway, NJ, USA).

### 2.4. Western Blot Analysis

Following radioactivity detection, the membranes were rehydrated in Tris buffer saline with 0.5% Tween 20 (0.5% TBST). Of the non-fat dry milk (Biorad), 5% was prepared in 0.1% TBST and was used to block non-specific reactions. The membrane was then probed by using antibodies against actin (Sigma Aldrich), eIF2α and phosphorylated eIF2α, ser51 (peIF2α) (Cell Signaling, Beverly, MA, USA) while aliquots from the same sample were used in another blot to detect virus protein synthesis using K95 polyclonal rabbit anti-IPNV [26]. This was followed by incubation with HRP-labeled secondary antibodies. The signal was developed using SuperSignal West Dura Extended Duration Substrate (Pierce) and detected using the Typhoon (GE healthcare). After the first detection, the membrane was washed twice with 0.1% TBST, stripped using Restore™ Plus buffer (Pierce, Rockford, IL, USA), washed twice as above, and re-probed with a new primary antibody and developed as already described.

### 2.5. Cell Viability Assay

Cell viability at different times post IPNV infection was determined by using CellTiter 96® AQueous One Solution Assay (Promega, Madison, WI, USA). The assay measures the reduction of a tetrazolium compound by the cells into a colored formazan product, which is directly correlated to the cell number. Confluent CHSE-214 cells seeded in 96-well plates (Corning, Life Science, Lowell, CA, USA) were infected with IPNV as described above or left uninfected. Cells were kept at 15 °C during the infection period. At 3, 12, 24 and 48 hpi, 20 µL of the CellTiter 96® AQueous One Solution reagent was added to the cells. Cells were incubated at 20 °C for 6 h and the absorbance at 490 nm was measured using a GENios microplate reader (Tecan, Männedorf, Switzerland). The viability was calculated and expressed as a percentage of the OD_490_ values obtained from the corresponding controls.

### 2.6. Assessment of Apoptosis by DNA Fragmentation

The fragmentation of DNA was assessed by using Apoptotic DNA Ladder Kit (Roche, Basel, Switzerland). Six-well plates containing confluent CHSE cells were used for this purpose. Cells were infected in quadruplicates with IPNV as described above while the remaining two wells were left uninfected as negative controls. Infected cells were sampled at 3, 12, 24 and 48 h post infection, while untreated cells were sampled at 48 h post infection. Cells were harvested by lysis using 400 µL binding/lysis buffer diluted v/v in PBS. Following incubation for 10 min, 100 µL isopropanol was added and mixed with the sample. The samples were then loaded in polypropylene tubes containing two layers of glass fiber fleece, washed using washing buffer, and finally eluted in 200 µL elution buffer. Samples were treated with 2 µg/mL DNase free RNase (Roche) before being analyzed by gel electrophoresis.

### 2.7. Caspase 3 Assay

The activity of caspase 3 in the cell lysates was assessed by using the Caspase-Glo^®^ 3/7 assay kit (Promega) following the protocol as described by the manufacturer. Briefly, CHSE-214 cells were seeded in white-walled 96-well plates (Corning) to a final density of 10^4^ cells/well and incubated for about 14 h. Cells were then infected with IPNV in triplicates sequentially as already described above. Duplicates of uninfected cells or cells treated with 2.5 μM staurosporine for 3, 12, 24 and 48 h were included as negative and positive controls, respectively. At the time of sampling, the volume of media in the wells was adjusted to 50 μL and equal volumes of Caspase-Glo^®^ 3/7 were added to each well. Cells were incubated at room temperature for 1 hour prior to bioluminescence reading (490 nm) using a GENios microplate reader (Tecan). The results were calculated by subtracting the mean values obtained from the control samples from the readings obtained from each well. The whole experiment was repeated twice.

### 2.8. Assessment of Apoptosis by Flow Cytometry

Six-well plates containing confluent CHSE cells were used in a similar setup as the one described in the section above. In addition, three parallel wells in a separate plate were treated with 2.5 µM staurosporine for 12, 24 or 48 h as positive controls of apoptosis. During each sampling, the supernatant was transferred to a 5 mL polystyrene round-bottomed tube (BD Biosciences, San Jose, CA, USA) while adherent cells were washed twice with PBS prior to trypsinization. Trypsinization was done for 5 min following which the reaction was stopped by adding fresh media. The trypsinized cells were then pooled with the original supernatant in the 5 mL polystyrene tubes. The cells were pelleted by centrifugation at 300× *g* for 10 min. The supernatant was then removed and cells were re-suspended in 100 µL Hepes buffer containing 2 µL fluorescein conjugated annexin-V staining reagent (Annexin-V-FLUOS Staining Kit, Roche). After incubating for 30 min, the volume was adjusted to 300 µL. To differentiate between apoptotic and necrotic cells, membrane permeability was assessed by adding propidium iodide (PI) (Sigma Aldrich), just before the analysis to a final concentration of 8 µg/mL. Flow cytometry was performed for 30,000 events using a BD FACSARIA^TM^ cell sorter (BD) while data analysis was performed using BD FACS DiVa Software, version 5.0.2 (BD).

The following parameters were measured to identify the apoptotic cells: (1) the area pulse of forward light scatter (FSC-A) *versus* side scatter (SSC-A), and (2) fluorescent intensities of FITC (filter 530/30) and PI (filter 630/30) upon excitation with 20 mW 488 nm laser. Cell aggregates were identified and excluded by using the width pulse of FSC-A *versus* area width of SSC-A. The whole experiment was repeated four times.

### 2.9. Quantitative Real-Time PCR Analysis

To assess the level of replication of IPNV as well as the expression of IFNα, Mx1 and PKR, real-time RT-qPCR was used. Cells were infected with IPNV as described above with the exception that this time three parallels were used per treatment. In addition, one parallel of cells was treated with IFNα for 4 days, as described previously, [27] as a control.

Total RNA was isolated by using the RNeasy Plus minikit (Qiagen, Hilden, Germany) according the manufacturer’s instructions and the concentration of RNA was determined by using the Nanodrop ND1000 (NanoDrop technologies, Wilmington, DE, USA). Of total RNA, 400 ng from each sample were used to synthesize cDNA using a Transcriptor first-strand cDNA synthesis kit (Roche) according to the manufacturer’s instructions. The cDNA was diluted five times and stored at −20 °C until required.

Quantitative PCR was performed in 96-well plates using the LightCycler 480 system (Roche). For each reaction, 2 µL cDNA was mixed with 10 pmol gene-specific primers and 10 µL LightCycler 480 SYBR green I master mix (Roche). The final concentration was adjusted to 20 µL using RNase-free water. The sequences of primers used in the reactions are provided in Table 1.

The cycling conditions for the PCR reactions were as follows: denaturation 94 °C for 10 s; annealing 60 °C for 10 s; elongation 72 °C for 10 s. The results were analyzed by the ∆∆CT relative quantification approach [28] using β-actin as reference gene. Graphs were drawn with the help of GraphPad Prism 5.0 (GraphPad Software Inc., San Diego, CA, USA).

**Table 1:** Primer sequences used for real-time PCR

Name	Primer sequence (5’-3’)	Genbank accession no.
Mx-Forward Mx-Reverse	TGCAACCACAGAGGCTTTGAA GGCTTGGTCAGGATGCCTAAT	U66475
B actin Forward B actin Reverse	CCAGTCCTGCTCACTGAGGC GGTCTCAAACATGATCTGGGTCA	AF012125
PKR Forward PKR Reverse	TGAACACAGCCAGAAGAACAA GACTACCGCCACATAACTCCA	EF523422.1
IPNv Forward IPNv Reverse	CAACAGGGTTCGACAAACCATAC TTGACGATGTCGGCGTTTC	
IFNα Forward IFNα Reverse	TGGGAGGAGATATCACAAAGC TCCCAGGTGACAGATTTCAT	NM_001123570.1

### 2.10. Statistical Analysis

For the caspase assay, two-way analysis of variance was used to test for differences between staurosporine-treated and infected cells followed by the Bonferroni test to compare the mean of each treatment to the mean of the RLU values obtained at 3 h. An Anova test was performed to assess reduction in protein synthesis and viability with a Dunnett *post hoc* test using GraphPad Prism 5.0 (GraphPad Software Inc.).

## 3. Results

### 3.1. Virus Replication in CHSE-214 Cells

**Figure 1:**
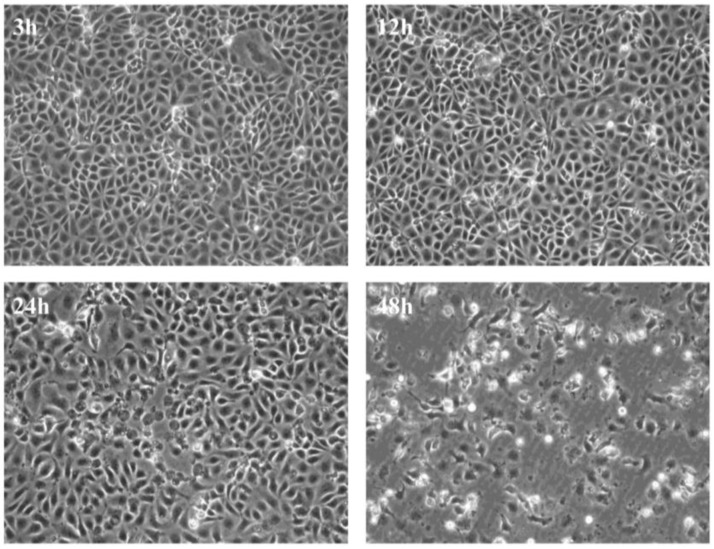
Cytopathic effects in CHSE cells**.** Development of cytopathic effect in CHSE-214 cells infected with recombinant strain rNVI-15R^b^ of infectious pancreatic necrosis virus.

Initially, we wanted to establish a system by which virus replication and its effect on cellular responses could be monitored over a short period of time thereby allowing, us to assess the effect of virus infection from the early replication stages until the endpoint in cell culture. We found that infection with 20 pfu/cell results in appearance of evident CPE by 48 h from the time of infection (Figure 1).

### 3.2. Virus Replication Results in Inhibition of Protein Synthesis

**Figure 2:**
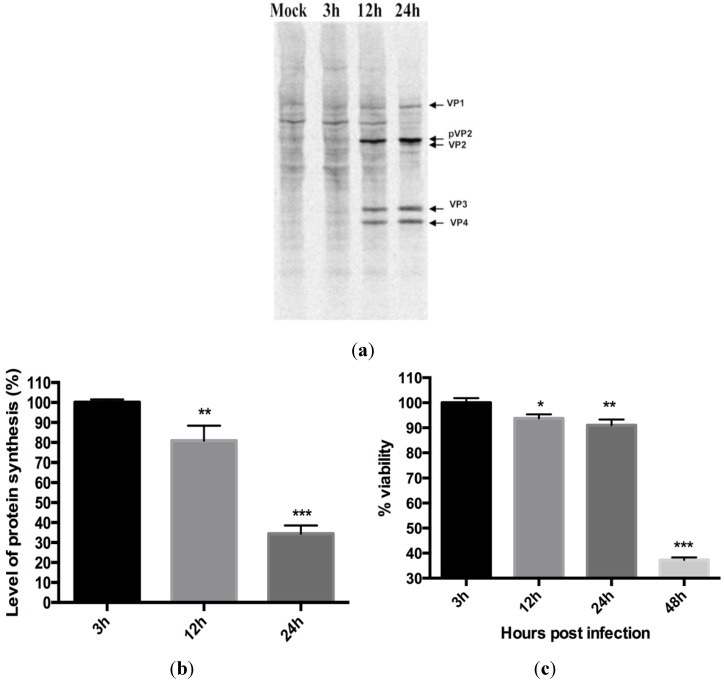
Infectious pancreatic necrosis virus induced inhibition of protein synthesis in CHSE 214 cells. Cells were first infected and, at indicated times, proteins synthesized were labeled using S^35^ Methionine and then harvested. (**a**) Prepared lysates were subjected to SDS-PAGE, blotted onto a PVDF membrane and autoradiographed in storage phosphor cassettes before analysis using the Typhoon. The numbers represent time in hours post infection; (**b**) Levels of protein synthesis expressed as percentages of the mock-infected cells after measurement of mean density protein amounts of three different bands using ImageQuant software (GE Healthcare). The results are representative of four independent experiments. *p < 0.05; **p < 0.01; (**c**) Cell viability post IPNV infection. Cells were infected with IPNV and cell viability was assayed at the indicated time post infection using CellTiter 96® AQueous One Solution assay (promega). The percent viability was calculated as described in the methodology section. Bars represent the average of 8 measurements taken from two independent infections ± SD. *p < 0.05, **p < 0.01, ***p < 0.001.

After establishing our infection model we examined protein synthesis in CHSE cells infected with IPNV at different times post infection (p.i.). At three hours p.i. (hpi), no difference in protein synthesis was observed between infected cells and uninfected controls (Figure 2a). At 12 and 24 hpi, however, a progressive reduction in protein synthesis was observed in infected cells, initially with a moderate reduction at 12 h (80% protein synthesis compared to uninfected controls; *p* < 0.01) followed by a marked reduction at 24 hpi, 65% reduction compared to uninfected controls (Figure 2b, *p* < 0.001). The loss of cell viability was 6% (*p* < 0.05) and 9% (*p* < 0.01), respectively, at these time points (Figure 2c), showing a pronounced reduction in protein synthesis with marginal reduction in viability.

### 3.3. Virus Replication Increases despite Inhibition of Host Protein Synthesis at Early Time Post Infection

**Figure 3:**
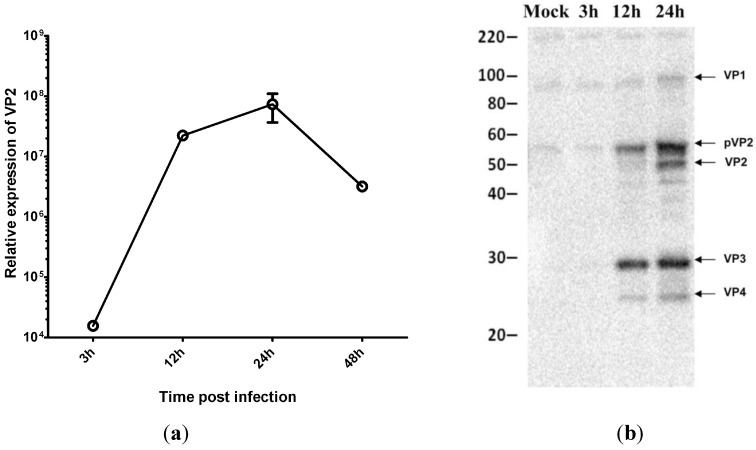
IPNV replication in virus-infected CHSE cells. (**a**) Relative expression of infectious pancreatic necrosis virus mRNA at different time post infection (n=3); (**b**) Western blot analysis for IPNV proteins synthesis at different times post infection.

Next we wanted to investigate whether inhibition of protein synthesis has any effect on virus replication. We first examined IPNV replication at different stages of infection by real-time RT-qPCR using primers specific for the virus VP2 protein. The results show that the viral mRNA levels increased rapidly in CHSE cells, peaking at 24 h (Figure 3a). Since production of new viruses can be inhibited at the translation level, we used Western blot as an additional tool to monitor the production of virus proteins. Our results showed that inhibition of protein synthesis had no effect on production of virus proteins (Figure 3b).

### 3.4. IPNV Infection of CHSE Cells Results in eIF2α Phosphorylation

**Figure 4:**
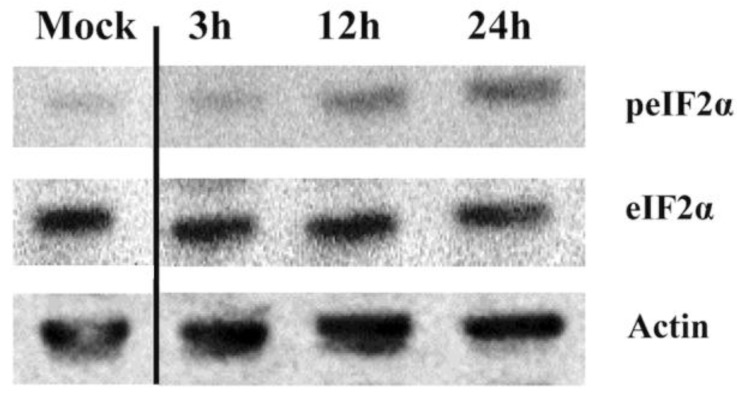
eIF2α phosphorylation in IPNV-infected CHSE-214 cells. CHSE-214 cells were infected with IPNV, cells were lysed at the indicated time points, and lysates were subjected to Western blot analysis using rabbit polyclonal antibodies against the phosphorylated form of eIF2α (Invitrogen). The blot is representative of a minimum of three independent observations. The numbers represent time in hours post infection.

Phosphorylation of eIF2α by different kinases is an important mechanism of protein synthesis regulation. In the present study, the onset of eIF2α phosphorylation was 12 hpi (Figure 4) and coincided with that of protein synthesis inhibition (Figure 2). At 24 h, the intensity of eIF2α phosphorylation increased (Figure 4) while there were not enough proteins left from the infected cells at 48 hpi to allow assessment.

### 3.5. The Effect of Protein Synthesis Inhibition on Type I IFN Response

**Figure 5:**
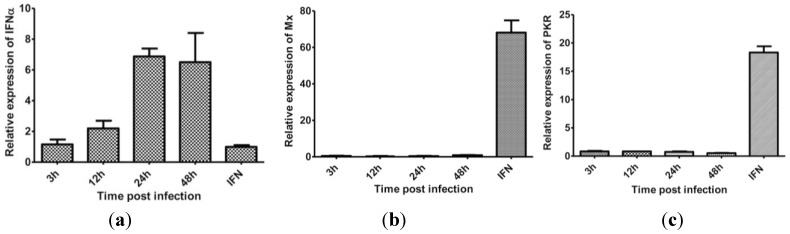
IFNα and Mx1 mRNA expression in IPNV-infected CHSE-214 cells. CHSE-214 cells were infected with IPNV, harvested at indicated time points, and IFNα (**a**), Mx1 (**b**) and PKR (**c**) mRNA expression were measured by real-time PCR. IFN is cells treated with recombinant IFNα for 4 days and used as positive control (n = 3).

Inhibition of protein synthesis is a global effect at cellular level and thus affects IFNα expression *per s*e and also downstream effector responses. Antibodies to salmon IFNα are not available to us and therefore we examined this effect by measuring IFNα mRNA levels. To estimate the production of IFNα at the protein level, an indirect approach using Mx1 and PKR mRNA expression, which comes down stream of IFNα signaling, was used. The results show that while IFNα mRNA gradually increased following infection (Figure 5a) and peaking at 24 hpi, Mx1 or PKR expression was not induced at any of the time points examined (3−48 hpi, Figure 5b,c). Our interpretation is that IPNV signals off a response in the cell that interferes with IFNα responses, either through a direct impact on IFNα levels or indirectly through downstream effects of IFNα.

### 3.6. IPNV Induces More Necrosis than Apoptosis at Early Time Post Infection

**Figure 6:**
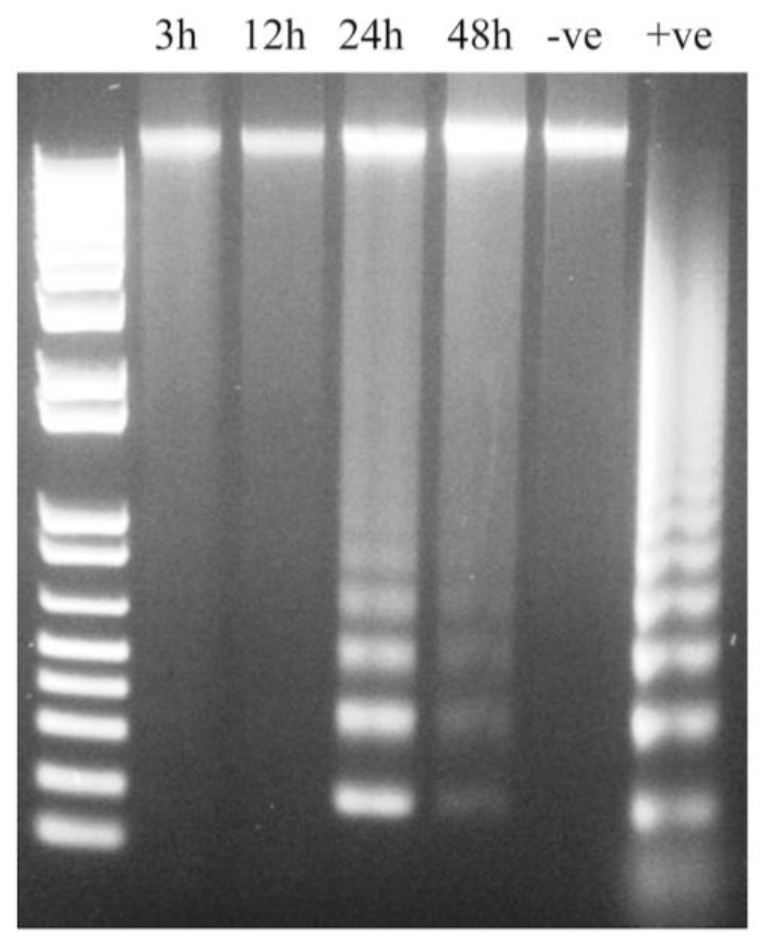
DNA laddering in IPNV-infected CHSE-214 cell lines. Cells were infected and harvested at the indicated time points. DNA was extracted using the Apoptotic DNA Ladder Kit (Roche) before being analyzed by gel electrophoresis. The result is representative of three independent experiments. –ve: uninfected cells; +ve: positive control.

**Figure 7:**
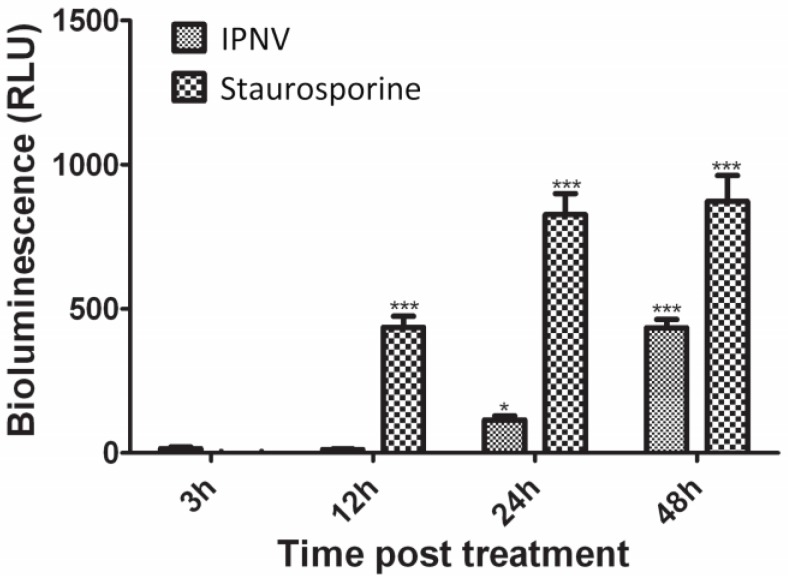
Caspase 3 activity in cells infected with infectious pancreatic necrosis virus. Cells were infected with IPNV or treated with 2.5 µM staurosporine (positive control) for the indicated periods. Uninfected and untreated cells were used as negative controls. Caspase 3 activity was assessed by measuring the bioluminescence resulting from cleavage of a luminogenic substrate using Caspase-Glo^®^ 3/7 assay kit (promega). The bars represent the relative luminescence units after subtracting the mean values obtained from the uninfected/untreated cells. Bars represent means of data obtained from two independent experiments each conducted using three parallels ± S.E.M. Statistical significance compared to cells treated at 3 h is indicated by asterisks; * = *p* < 0.05; *** = *p* < 0.001.

**Figure 8:**
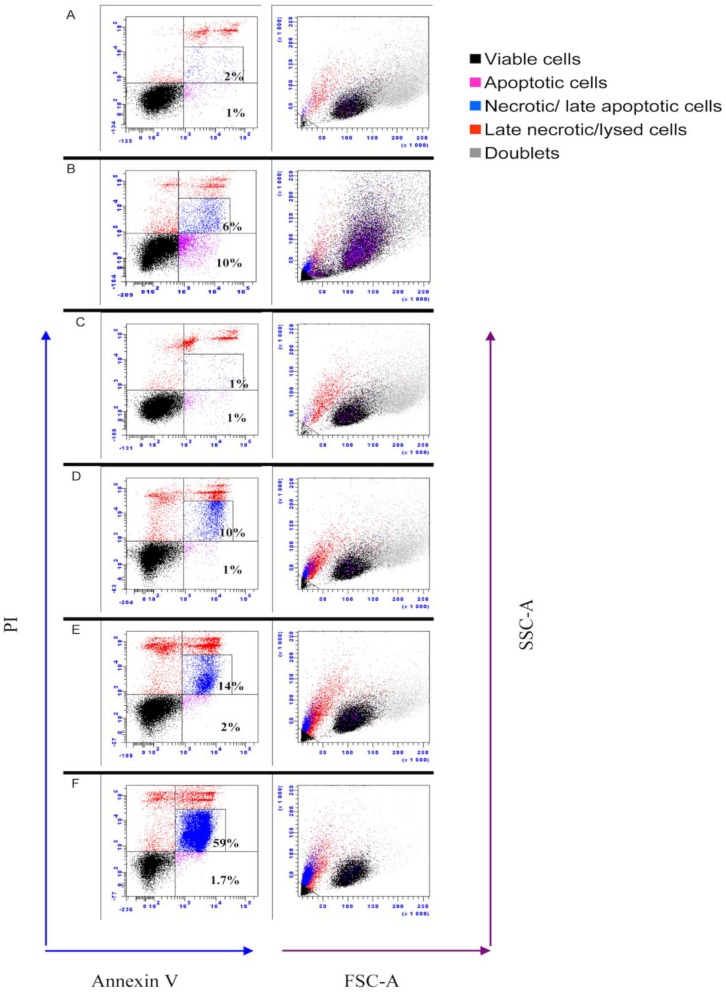
Flow cytometric analysis of infectious pancreatic necrosis virus-infected CHSE-214 cells. Cells were stained with annexin V and propidium iodide (PI) prior to analysis at prescribed time points. **A** = non infected cells; **B** = cells treated with staurosporine for 48 h to induce apoptosis; **C-F** = IPNV-infected cells for 3, 12, 24 and 48 h, respectively. Necrotic cells (blue) have low forward (FSC-A) and side scatter (SSC-A) values. Results provided are representative of at least four independent observations.

Previous studies have shown that IPNV strains of serotype Ab induce apoptosis at early time post infection [29]. Since apoptotic cells exhibit decreased protein synthesis, this could serve as an explanation to the reduced protein synthesis referred above and therefore we needed to understand what role apoptosis played with regard to reduced protein synthesis. First, a DNA laddering assay was used. At 3 and 12 hpi, no DNA fragmentation was observed; however, at 24 and 48 hpi, fragmentation of DNA was visible (Figure 6), thus suggesting involvement of apoptosis. Since apoptosis-like DNA laddering is known to occur also as a result of necrosis [30,31], DNA laddering alone cannot fully distinguish between apoptosis and necrosis. The caspase 3 assay and flow cytometry were therefore used to validate these findings. In IPNV-infected cells, a significant increase in caspase 3 activity was observed both at 24 (*p <* 0.05) and 48 hpi (*p <* 0.001) (Figure 7), in agreement with the DNA laddering (Figure 6). Caspase 3 activity in staurosporine-treated cells (positive controls) was observed from 12 h post treatment and onwards (Figure 7). For flow cytometry, we used double-staining for annexin V (AV) and Propidium Iodide (PI) to represent apoptosis and necrosis, respectively. Cells that are AV^+^/PI^-^ are apoptotic while AV^+^/PI^+^ or AV^-^/PI^+^ are necrotic [32]. IPNV-infected cells showed a marked increase for AV^+^/PI^+^ staining from 12 h onwards (Figure 8; from 10 to 59% double-positive cells), demonstrating that the cells’ membrane integrity was compromised. On the other hand, AV^+^/PI^-^ stained cells remained low, and increased from 1 to 2% by 24 h and 1.7% by 48 h.

## 4. Discussion

Here we show for the first time that infectious pancreatic necrosis virus induces inhibition of protein synthesis in the permissive cell line, CHSE. The inhibition involves phosphorylation of eIF2α (from 12 hpi and onwards). While apoptosis plays less of a role in protein synthesis inhibition, and DNA laddering is seen by 24 hpi, the highest percentage of apoptotic by 24 hpi is 2% by flow cytometry. Despite cellular protein shutdown, virus replication peaks at 24 hpi and a drop in cell viability is seen earliest at 48 hpi, coinciding with the loss of membrane integrity (by flow cytometry). Because inhibition of protein synthesis did not hinder virus production, we propose this is a strategy of the virus to circumvent IFNα-induced cellular, antiviral mechanisms and to induce cell lysis in order to facilitate its release from infected cells.

Phosphorylation of eIF2α and the subsequent inhibition of protein synthesis are important host defensive mechanisms to limit the replication of RNA viruses [33]. eIF2α phosphorylation following IPNV infection (Figure 4) is consistent with a previous report [21] and results in inhibition of protein synthesis in CHSE-214 cells. Two kinases have been shown to play roles in eIF2α phosphorylation during virus infections in higher vertebrates, namely the PKR and PERK [1,3]. Fish possess an additional kinase named Z-DNA binding protein kinase (PKZ) and Atlantic salmon PKZ has been shown to phosphorylate eIF2α in response to Z-DNA, but its activation by Z-dsRNA has not yet been demonstrated [34]. Antibodies against PKR, PERK, or their phosphorylated forms are not available for salmonids at the moment. It was therefore difficult for us to further study and demonstrate the mechanisms by which eIF2α is induced. Both PKR and PERK have been suggested to be involved in inducing cell death following IPNV infection [35]. Indeed, high virus replication and protein synthesis as observed in this study and accumulation of folded proteins may have led to PERK activation as a consequence of ER stress followed by eIF2α phosphorylation. However, ER-stress response has only been shown to occur during infection with enveloped viruses [36]. PKR, on the other hand, is found in the cytoplasm where IPNV replication occurs. In fact, IPN viral RNA is exposed in the cytoplasm and can be detected in infected cells during replication through various receptors [37]. Despite PKR not being found as upregulated, basal levels of PKR could be responsible for eIF2α phosphorylation during IPNV infection. However, additional studies should be performed to elucidate this in detail.

Contradicting reports have been published regarding the occurrence of apoptosis following IPNV infections [29,38,39,40,41]. Since eIF2α has previously been implicated in decreased protein synthesis of apoptotic cells [42], we needed to understand to what extent apoptosis *per se* contributed to protein shutdown. All apoptosis assays used in the present study, namely DNA laddering, caspase 3 and flow cytometry, showed that apoptosis occurred from 24 h onwards (Figure 6, Figure 7 and Figure 8), but the fraction of apoptotic cells is very small, based on flow cytometry (Figure 8), which is consistent with our earlier results [41]. Intriguingly, loss of membrane integrity as detected by PI staining (Figure 8) appears earlier than apoptosis, similar to the findings of some [38] but at variance with results of others [29,39,41]. The differences in apoptosis/necrosis profiles between our findings and other researchers [29,38,39,40,41] demonstrate that different IPNV isolates are associated with variable apoptotic/necrotic characteristics. Care should therefore be taken not to generalize results beyond isolates or geno-groups.

Perforation of cell membranes and the subsequent changes in membrane permeability in virus-infected cells may occur at early stages due to virus entry, during virus maturation or late stages of virus replication resulting in cell lysis and virus release [43]. It has also been suggested that permeability changes may be essential for cells to switch from synthesis of cell proteins to virus proteins [43]. Forming of pores during the entry process is sensitive to the concentration of extracellular Ca^2+^ and can be inhibited by higher concentrations [43]. In the present study, changes in membrane permeability started at middle stages of virus replication (12 hpi) and reached the peak at late stages of virus replication with the onset of cell lysis. No change in membrane permeability was detected at early stages. This suggests that changes in membrane permeability during IPNV infection may be essential for virus replication and/or release. This is similar to what has been found for infectious bursal disease (IBDV), another member of the family *Birnaviridae* [44] where no virus was rescued after disrupting the pore forming mechanism by mutagenesis. Pore formation by IBDV could also be inhibited by increasing Ca^2+^ concentrations. The ability of IPNV to perforate cell membranes is yet to be demonstrated. However, high extracellular Ca^2+^ was shown to decrease plaque formation in CHSE cells while the opposite was observed when extracellular Ca^2+^ was decreased or with Ca^2+^ blockers [45].

To overcome the effect of protein shutdown, several species of RNA viruses have evolved a cap-independent (eIF2α-independent) mechanism of initiation of translation. For example, viruses belonging to the family *Picornaviridae* possess internal ribosome entry sites (IRES) that enable them to preferentially translate their proteins in a cap-independent manner [46]. Hantaviruses have a unique cap-independent mechanism by which they utilize their nucleocapsid protein to replace all or part of the cap-binding complex [47]. For the *Birnaviridae* family, there is limited information on the initiation of translation and replication and it is not known if they possess a ribosome entry site (IRES) of normal (*i.e*., >300 nt) length or another mechanism that is needed to recruit host cell-encoded initiation factors [48]. We found that eIF2α phosphorylation was induced as early as 12 hpi and the phosphorylation levels continued to increase for the duration of the infection cycle accompanied by marked inhibition of cellular protein synthesis (Figure 2 and Figure 7). On the other hand, virus protein synthesis increased during the time of host protein inhibition (Figure 3b). What mechanisms are used by IPNV to initiate translation under these circumstances remains unresolved and additional studies are needed to understand this in more detail.

Inhibition of protein synthesis is a double-edged sword; whereas it is an important defensive tool for the host cell [49], it nevertheless limits the antiviral effects downstream of interferon responses, and can thus be a survival strategy for the virus [50]. Previous studies show that IPNV infections do not suppress IFN production but interfere with downstream signaling [51]. Skjesol *et al.* [52] confirmed this and further demonstrated that IPNV infections are able to attenuate IFN induced responses even when the cells were pretreated with IFN for 4 h. Herein we show that inhibition of protein synthesis was induced while virus replication was at its highest (Figure 2 and Figure 3) and with a parallel increase of IFNα mRNA expression (Figure 8a). Regardless, neither Mx1 nor PKR mRNA was induced and from this we interpret the results to imply that IPNV induced inhibition of protein synthesis may serve as a strategy for the virus to evade or attenuate interferon-induced anti-viral responses and/or to facilitate the release of viral progeny.

Inhibition of protein synthesis post IPNV infection found here is in contrast with previous reports where no protein inhibition was observed following infection of RTG cells although host DNA synthesis was inhibited [19,20,53]. The reasons for this are not clear but there are several differences between the present study and the previous ones. We used CHSE cells instead of RTG-2 cells but we consider it unlikely that this would account for the particular differences observed since the isolate used has also the ability to inhibit protein synthesis in RTG-2 cells [22]. Moreover, the virus isolates used in previous studies are different from ours; they used the so-called Dry Mills strain [19,20], which is closely related to AF343571 and isolate VR-299 [53]. Both isolates belong to the North American West Buxton (WB) serotype (serotype A1); we used an Sp serotype (serotype A2) that is antigenically and pathogenically distant from the WB isolates [54] in the present study. In addition to this, there are differences in the multiplicity of infections used or the time of sampling. While Nicholson and Lothrop used very high MOI, 100TCID_50_/Cell [19,20], Dobos used similar MOI to ours, 20 PFU/cell, but the last sample was obtained at 12 hpi which in our study was found to be the start of protein inhibition [53].
